# Altered nociceptive behavior and emotional contagion of pain in mouse models of autism

**DOI:** 10.1111/gbb.12778

**Published:** 2021-11-23

**Authors:** Loren J. Martin, Sandra J. Poulson, Emma Mannan, Sivaani Sivaselvachandran, Moonjeong Cho, Fatima Setak, Claire Chan

**Affiliations:** ^1^ Department of Psychology University of Toronto Mississauga Mississauga Ontario Canada; ^2^ Cell and Systems Biology University of Toronto Toronto Ontario Canada

**Keywords:** autism, BTBR, emotional contagion, FMR1, formalin, inflammatory, mechanical sensitivity, mouse, pain, sensory

## Abstract

Individuals with autism spectrum disorder (ASD) have altered sensory processing but may ineffectively communicate their experiences. Here, we used a battery of nociceptive behavioral tests to assess sensory alterations in two commonly used mouse models of ASD, BTBR T^+^ *Itpr3* ^*tf*^/J (BTBR), and fragile‐X mental retardation‐1 knockout (*Fmr1*‐KO) mice. We also asked whether emotional contagion, a primitive form of empathy, was altered in BTBR and *Fmr1* KO mice when experiencing pain with a social partner. BTBR mice demonstrated mixed nociceptive responses with hyporesponsivity to mechanical/thermal stimuli and intraplantar injections of formalin and capsaicin while displaying hypersensitivity on the acetic acid test. *Fmr1*‐KO mice were hyposensitive to mechanical stimuli and intraplantar injections of capsaicin and formalin. BTBR and *Fmr1*‐KO mice developed significantly less mechanical allodynia following intraplantar injections of complete Freund's adjuvant, while BTBR mice developed slightly more thermal hyperalgesia. Finally, as measured by the formalin and acetic acid writhing tests, BTBR and *Fmr1‐*KO mice did not show emotional contagion of pain. In sum, our findings indicate that depending on the sensation, pain responses may be mixed, which reflects findings in ASD individuals.

## Graphical abstract

BTBR T+ Itpr3tf/J mice, an idiopathic model of autism spectrum disorder demonstrate mixed pain responses when tested on a battery of pain tests. Fmr1‐KO mice, a monogenic model of autism spectrum disorder lack overall sensitization when tested on a battery of pain tests. Both mouse strains do not show social pain contagion responses in the presence of another mouse.

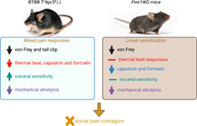

## 1 INTRODUCTION

Autism spectrum disorders (ASD) are a highly prevalent class of neurodevelopmental disorders characterized by social and communicative impairments and restricted behavior.^1^ In addition to the core social deficits, a growing body of work also implicates abnormal sensory responses as a critical symptom of ASD in multiple forms and across modalities.^2^, ^3^, ^4^ Tactile hypersensitivity appears to be especially common, appearing in up to 95% of cases,^5^ and often presents itself as defensiveness or avoidance and interferes with social behavior that involves interpersonal touch.^6^ While as many as 44% of ASD patients engage in self‐injurious behaviors such as head banging, hair pulling, skin picking, and scratching, the experience of pain remains poorly understood in ASD individuals.^7^

Evaluation of sensory phenotypes has shown that ASD individuals display altered nociceptive behavior but depending on the type of noxious stimulation, pain responses may increase or decrease.^4^ For instance, adolescents with ASD exhibit decreased thermal sensitivity,^8^ while high functioning ASD children reportedly have increased pressure and mechanical sensitivity compared to typically developed children.^9^ In addition, the lack of communication skills in ASD may not allow these individuals to communicate the degree and severity of pain experienced effectively. At the same time, ASD individuals may also not fully understand pain signals from others within their social environment.^10^

Animal models have proven invaluable for investigating the core features and genetic bases of ASD,^11^ with some recent studies also investigating altered sensory function.^12^ For instance, mutations in the major *SHANK* isoforms (SH and multiple ankyrin repeat domains), a family of scaffolding proteins, have been associated with ASD in humans.^13^, ^14^, ^15^ Mice harboring mutations of the *Shank2* gene exhibit social impairments,^16^ while *Shank3* null mice display decreased social communication as measured by ultrasonic vocalizations but normal levels of sociability.^17^ Null deletion of *Shank2* leads to reduced mechanical and thermal pain sensitivity,^18^ while *Shank1* and *Shank3* null mice display normal thermal pain responses^19^, ^20^; however, global or sensory neuron‐specific deletion of *Shank3* impairs heat hyperalgesia.^20^ Heterozygous loss of either *Tsc1* or *Tsc2* (tuberous sclerosis complex), two genes associated with ASD in humans,^21^ leads to social deficits, repetitive rearing, and learning and memory impairment in mice, but intact sensory function.^22^ There is also evidence that deletion of *Mecp2* (methyl CpG binding protein 2), a model of human Rett syndrome commonly classified as an ASD, is associated with decreased heat responsiveness,^23^ while also linked to increased mechanical sensitivity.^24^

Fragile X syndrome, another common syndromic form of ASD, is associated with pervasive intellectual disability, repetitive behaviors, social deficits, and increased anxiety.^25^ Null deletion of the *Fmr1* gene, which encodes for the fragile X mental retardation 1 protein (FMRP), yields social deficits^26^ and decreased responses to inflammatory and neuropathic pain,^27^ but hypersensitivity to tactile stimuli.^28^ In another study, *Fmr1* knockout mice and BTBR T^+^ *Itpr3* ^*tf*^/J (BTBR) mice, another model of ASD, showed hyporesponsiveness to thermal stimuli and hyperresponsiveness to intraperitoneal injections of acetic acid.^29^ The BTBR mouse model is interesting because several ASD‐relevant mRNAs are altered, including *Neurexin*‐1 and *Homer31*, which bind^30^ to *Shank1* and *Shank3* and has been linked to over activation of the mechanistic target of rapamycin (mTOR) pathway as observed in syndromic forms of ASD such as tuberous sclerosis complex and fragile X syndrome.^31^

In the current paper, we assessed nociceptive behaviors of BTBR, an idiopathic model of ASD, and *Fmr1* null‐mutant mice (*Fmr1*‐KO), a monogenic model of ASD using a battery of innocuous and noxious pain tests. We also studied the development of chronic inflammatory pain and whether hypersensitivity was altered in these models of ASD. Finally, given that BTBR, and *Fmr1*‐KO mice have impaired social behavior, we assessed whether the emotional contagion of pain was impaired in these mice. Emotional contagion—a primitive form of empathy—enhances pain behavior when familiar mice are tested together^32^, ^33^; however, it remains unclear whether the emotional contagion of pain is altered in ASD mouse model.

## 2 METHODS

### 2.1 Mice

All experiments were performed on young (6–12 weeks) adult male, C57BL/6J, BTBR T^+^ Itpr3^tf^/J (Jax stock, 002282) or *Fmr1*‐KO mice (*C57BL6/J background*, Jax stock, 003025) originally purchased from Jackson Laboratories. Mice were bred in‐house for several generations at our animal facility at the University of Toronto Mississauga. For some experiments, *Fmr1*‐KO mice (*C57BL6/J background*) were generously provided by Dr. David Hampson (University of Toronto). All mice were housed with the same sex in groups of four mice per cage, maintained in a temperature‐controlled (20 ± 1°C) environment with 12:12‐h light:dark cycle with access to food (Harlan Teklad 8604) and water ad libitum. Experiments were conducted only during the light period, and mice were habituated to the testing environment for at least 15 min in every assay before testing commenced. All procedures were approved by the University of Toronto animal care committee and in accordance with the Canadian Council on Animal Care.

### 2.2 von Frey test

An automated von Frey test (Ugo Basile Dynamic Plantar Aesthesiometer) was used to assess mechanical nociceptive thresholds. Mice were placed in custom‐constructed Plexiglas cubicles (6.3 × 5.5 × 10 cm) on a perforated metal floor and allowed to habituate for 1 h before testing. A blunt probe was raised toward the plantar surface of the hind paw, upon which pressure was gradually increased until the mouse withdrew its hind paw; the maximal pressure displayed at that point was then recorded. The average of three trials per mouse per paw was used as the measure of mechanical sensitivity.

### 2.3 Tail clip

A small alligator clip (force, 700×*g*) was applied at 1 cm from the base of the tail as we have previously performed.^34^ The latency to attack/bite the clip was measured to the nearest 0.1 s. Mice were only tested once on the tail clip test.

### 2.4 Cold plantar

We followed the procedure as described in Brenner, Golden, Gereau^35^ to measure cold sensation. Powdered dry ice was packed into a modified syringe, and the open end of the syringe was held against a glass surface while depressing the plunger to form a dry ice pellet that was extended past the end of the syringe and pressed to the glass underneath the hind paw using light, but consistent pressure applied to the syringe plunger. Care was taken to ensure that the hind paw was touching the glass surface. Latency to withdraw the hind paw from the stimulus was measured to the nearest 0.1 s. The average of three trials per mouse per paw was used as the measure of cold sensitivity.

### 2.5 Radiant heat paw‐withdrawal test

Mice were placed on a glass floor within small Plexiglas cubicles (9 × 5 × 5 cm high). Following habituation, a focused high‐intensity projector lamp beam was shone from below onto the mid‐plantar surface of the hind paw.^36^ The radiant heat device (IITC Model 336) was set to 20% active intensity. Latency to withdraw the hind paw from the stimulus was measured to the nearest 0.1 s. The average of three trials per mouse per paw was used as the measure of heat sensitivity.

### 2.6 Hotplate

Mice were placed into a clear Plexiglas cylinder atop a hotplate (Columbus Instruments) maintained at 50°C. The latency to lick or shake either hind paw was measured to the nearest 0.1 s. Mice were only tested once on the hotplate.

### 2.7 Capsaicin

Mice were placed on a glass floor within Plexiglas cylinders (30 cm high; 30 cm diameter) and allowed to habituate for 15 min. Mice then received a subcutaneous injection of capsaicin (2.5 μg; Sigma) into the plantar left hind paw (20 μl) and were digitally videotaped for 10 min. Video files were later scored for the total duration (s) of licking/biting of the injected paw.

### 2.8 Formalin test

Formalin injection produces a biphasic response: an acute, nociceptive “early” phase and a tonic, inflammatory “late” phase, separated by a quiescent period in which there is reduced pain behavior.^37^ Mice were placed on a glass platform within Plexiglas cylinders (30 cm high; 30 cm diameter) and habituated. Then, 20 μl of 2.5% formalin was injected intraplantar into the left hind paw using a 100‐μl microsyringe with a 30‐gauge needle. Behavior was video recorded for 60 min and coded offline, where the first 10 s of every minute was scored for the presence of licking/biting (positive sample) of the left hind paw. The early phase was defined as the percentage of positive samples during the first 0–10 min post‐injection of formalin; the late phase was the percentage of positive samples during the 10–60 min post‐injection period. The percentage of positive samples was then calculated and binned into 5 min intervals. For the social modulation experiments, mice were tested either singly (alone) or with a social partner (either a cagemate or stranger). For the social condition, both mice received formalin injections.

### 2.9 Acetic acid

Mice were habituated for at least 30 min to an observation chamber (15 cm diameter; 22.5 cm high), placed atop a glass surface suspended over high‐resolution video cameras. Mice were injected intraperitoneally (10 ml/kg) with 0.9% acetic acid and videotaped digitally for 30 min after the injection. The videotapes were later scored offline by a different experimenter who was blind to experimental details. Video files were coded for the number of lengthwise constrictions of the abdominal musculature (“writhes”) using a sampling procedure (1 sample every 20 s) as we have previously performed.^32^ For the social modulation of pain studies, mice were tested as described for the formalin experiments, the only difference being that acetic acid was used as the pain stimulus.

### 2.10 Complete Freund's adjuvant

Complete Freund's adjuvant (CFA; 50%; Sigma) was injected subcutaneously in a volume of 20 μl into the left plantar hind paw using a 100‐μl microsyringe with a 30‐gauge needle. Mice were tested for radiant heat paw withdrawal or sensitivity to von Frey filaments of both hind paws as described above, before, 3‐, 7‐ or 10 days post‐CFA injection. The percentage of allodynia/hyperalgesia for Day 3 was calculated as a function of baseline (i.e., decrease from baseline threshold) and reported as percentage change.

### 2.11 Three chamber test

We followed the experimental protocol described by Yang et al.^38^ and as we have previously performed.^39^ Briefly, we habituated test mice to the center of the three chambered apparatus for 10 min. Following initial habituation, mice were allowed to freely explore all chambers for an additional 10 min (baseline). A single naive mouse was then placed in an inverted wire pencil cup in one side chamber (in a counterbalanced fashion). These “stimulus” mice were previously habituated to the pencil cup to reduce excessive movement while in the cup. Test mice were videotaped for 10 min in the presence of the stimulus mice. The total time spent in each side chamber (one containing the stimulus mouse and the other a novel object) was coded by a blinded experimenter.

### 2.12 Statistical analysis

Data were analyzed by a 2‐tailed Student's *t*‐test (unless otherwise indicated) or two‐way ANOVA for CFA and pain contagion experiments, followed by Tukey's honest significant difference (HSD) post hoc tests. For CFA time‐course data, we conducted post hoc testing between mouse strains at each time point. For the pain contagion experiments, post hoc testing was conducted within strain and comparisons between the alone, cagemate, and stranger conditions were made. A *p*‐value of less than 0.05 was used to determine statistical significance. All data were analyzed using SPSS v 27.

## 3 RESULTS

### 3.1 Nociceptive sensitivity of BTBR mice on multiple pain modalities

**FIGURE 1:**
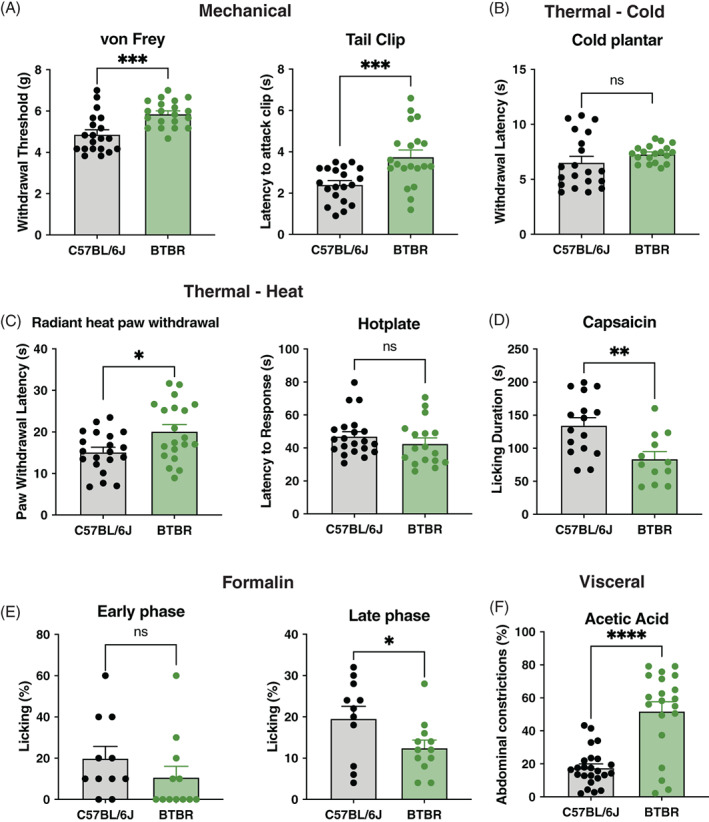
Behavioral battery of nociceptive tests in C57BL/6J and BTBR mice. (A) BTBR mice (*n* = 20) had higher mechanical thresholds on the von Frey and tail clip test than C57BL/6J (*n* = 20). (B) C57BL/6J (*n* = 20) and BTBR mice (*n* = 20) do not differ on the cold plantar assay. (C) BTBR mice (*n* = 20) have longer thermal latencies (decreased sensitivity) on the radiant heat paw withdrawal test but were not different when compared to C57BL/6J mice (*n* = 20) on the hotplate test. (D) BTBR mice (*n* = 12) display less licking behavior as measured by cumulative duration following plantar hind paw injection of capsaicin (2.5 μg) compared with C57BL/6J (*n* = 16 mice). (E) Graphs show early phase (0–10 min post‐injection) and late phase (10–60 min post‐injection) nocifensive behavior, respectively, after 2.5% formalin injection into the plantar hind paw for C57BL/6J (*n* = 11) and BTBR (*n* = 12) mice. (F) BTBR mice (*n* = 20) display significantly more abdominal constrictions than C57BL/6J mice (*n* = 24) following an intraperitoneal injection of acetic acid (0.9%). Bars in all graphs represent mean ± SEM; *p < 0.05, **p < 0.01, ***p < 0.0001

To study the nociceptive sensitivity of BTBR mice, we assessed multiple sensations, including mechanical, thermal, and chemical. BTBR mice exhibited a robust increase in mechanical thresholds measured using the von Frey and tail clip tests (von Frey: *t*_38_ = 3.91, *p* < 0.001; tail clip: *t*_38_ = 3.63, *p* < 0.001, Figure 1A). Cold sensitivity was unaffected; however, thermal heat thresholds were increased as measured by the radiant heat paw withdrawal, but not the hot plate test (cold plantar: *t*_38_ = 1.635, *p* = 0.11; radiant heat: *t*_38_ = 5.72, *p* = 0.014; hot plate: *t*_38_ = 1.04, *p* = 0.3, Figure 1B,C). Assessment of nocifensive behavior revealed that BTBR mice had reduced overall licking duration following intraplantar injection of capsaicin and decreased sensitivity on the late phase of the formalin test (capsaicin: *t*_26_ = 3.192, *p* < 0.01; early phase formalin: *t*_21_ = 1.179, *p* = 0.25; late phase formalin: *t*_21_ = 2.088, *p* = 0.04, Figure 1D,E). Interestingly, BTBR mice had increased visceral sensitivity on the acetic acid abdominal constriction assay (*t*_42_ = 6.07, *p* = 0.001, Figure 1F).

**FIGURE 2:**
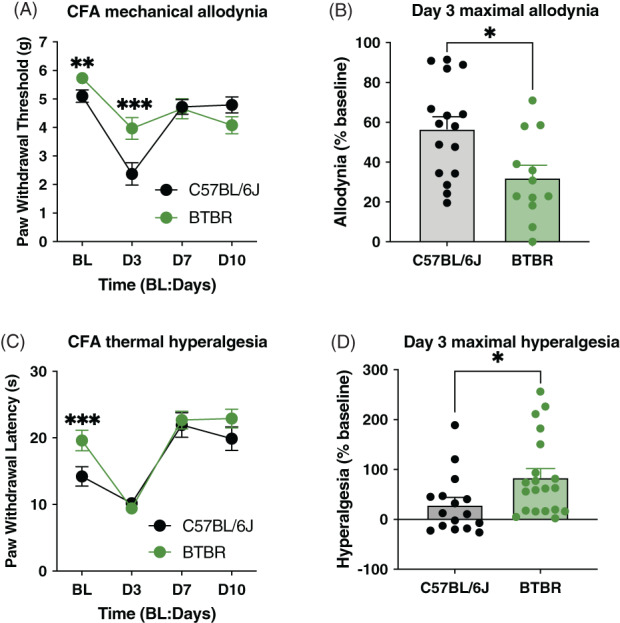
BTBR mice show altered nociceptive response in chronic inflammatory pain. (A) Basal (BL) mechanical withdrawal thresholds (g) were measured using the von Frey test followed by an intraplantar hind paw injection of complete Freund's adjuvant (CFA). BTBR (*n* = 12) display higher mechanical thresholds during BL and on Day 3 (D3) post‐CFA injection compared with C57BL/6J (*n* = 16) mice. Mechanical thresholds were not different between the strains on Day 7 (D7) or Day 10 (D10). (B) BTBR mice display less allodynia when von Frey thresholds on D3 are normalized to BL thresholds for each mouse. (C) BL thermal withdrawal latencies (s) were measured using the radiant heat paw withdrawal followed by an intraplantar hind paw injection of CFA. BTBR (*n* = 16) and C57BL/6J (*n* = 20) mice do not differ for thermal thresholds between the strains on D3, D7, or D10 post‐CFA injection. (D) BTBR mice display greater hyperalgesia when thermal thresholds on D3 are normalized to BL thresholds for each mouse. Symbols or bars in all graphs represent mean ± SEM; *p < 0.05, **p < 0.01, ***p < 0.0001

After characterizing acute nociceptive responses, we studied whether CFA‐induced mechanical and thermal hypersensitivity was altered in BTBR mice. BTBR mice had higher von Frey thresholds during baseline testing with mechanical sensitivity significantly reduced following CFA injection (two‐way ANOVA, main effect of strain: *F*_1,26_ = 1.243, *p* = 0.28; main effect of time [RM]: *F*_3,78_ = 30.128, *p* < 0.001; strain x time interaction: *F*_3,78_ = 8.27, *p* < 0.001; Figure 2A). As there were baseline differences between BTBR and C57BL/6J, we calculated the percentage of maximal allodynia on day 3, confirming that BTBR mice had less CFA‐induced mechanical sensitivity (*t*_26_ = 2.76, *p* = 0.01, Figure 2B). BTBR mice were less sensitive during baseline thermal measurements (two‐way ANOVA, main effect of strain: *F*_1,34_ = 4.11, *p* = 0.05; main effect of time [RM]: *F*_3,102_ = 35.75, *p* < 0.001; strain x time interaction: *F*_3,102_ = 1.92, *p* = 0.13; Figure 2C); however, CFA‐induced thermal hyperalgesia was more prominent in this strain (*t*_34_ = 2.313, *p* = 0.03; Figure 2D).

### 3.2 Nociceptive sensitivity of *Fmr1* knockout mice on multiple pain modalities

**FIGURE 3:**
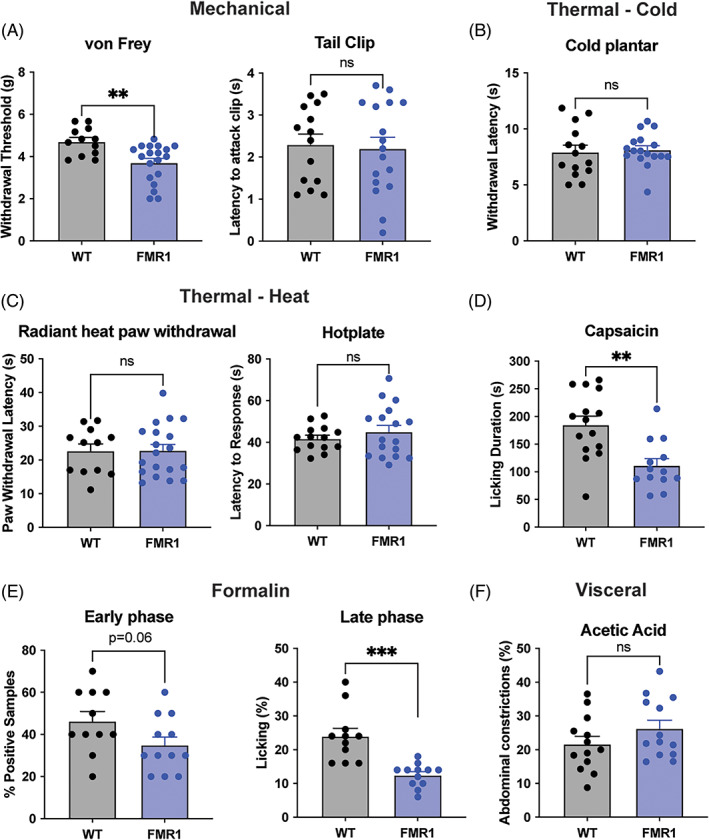
Behavioral battery of nociceptive tests in WT and *Fmr1*‐KO mice. A. *Fmr1*‐KO (*n* = 19) had lower mechanical thresholds on the von Frey but not tail clip test than WT (*n* = 12) mice. (B) WT (*n* = 14) and *Fmr1*‐KO (*n* = 17) mice do not differ on the cold plantar assay. (C) WT and *Fmr1*‐KO mice do not differ on the radiant heat paw withdrawal (*WT*, *n* = 12; *Fmr1*‐KO, *n* = 19) or the hotplate test (*WT*, *n* = 14; *Fmr1*‐KO, *n* = 17). (D) *Fmr1*‐KO mice (*n* = 13) display less licking behavior as measured by cumulative duration following plantar hind paw injection of capsaicin (2.5 μg) compared with WT (*n* = 15) mice. (E) Graphs show early phase (0–10 min post‐injection) and late phase (10–60 min post‐injection) nocifensive behavior, respectively, after 2.5% formalin injection into the plantar hind paw for WT (*n* = 11) and *Fmr1*‐KO (*n* = 12) mice. (F) WT (*n* = 13) and *Fmr1*‐KO (*n* = 13) mice do not exhibit differences in abdominal constrictions following an intraperitoneal injection of acetic acid (0.9%). Bars in all graphs represent mean ± SEM difference; **p < 0.01, ***p < 0.0001

*Fmr1‐KO* mice have been used as a monogenic model of ASD.^26^ Thus, we examined their nociceptive behavior using the same battery of tests as performed for the BTBR model. *Fmr1* knockout mice had significantly lower mechanical thresholds on the von Frey, but not the tail clip test (von Frey: *t*_29_ = 3.29, *p* = 0.002; tail clip: *t*_29_ = 0.26, *p* = 0.79; Figure 3A), whereas their thermal sensitivity was unaffected in the cold plantar, radiant heat paw‐withdrawal and hotplate tests (cold plantar: *t*_29_ = 0.31, *p* = 0.76; radiant heat: *t*_29_ = 0.02, *p* = 0.98; hotplate: *t*_29_ = 0.92, *p* = 0.36, Figure 3B,C). *Fmr1*‐ KO mice displayed less nocifensive behavior on the capsaicin test and during the formalin assay (capsaicin: *t*_26_ = 3.64, *p* = 0.001; early phase formalin: *t*_21_ = 1.94, *p* = 0.06; late phase formalin: *t*_21_ = 4.62, *p* < 0.001; Figure 3D,E **)** but were not different from WT controls on the acetic acid test (*t*_24_ = 1.37, *p* = 0.18; Figure 3F).

**FIGURE 4:**
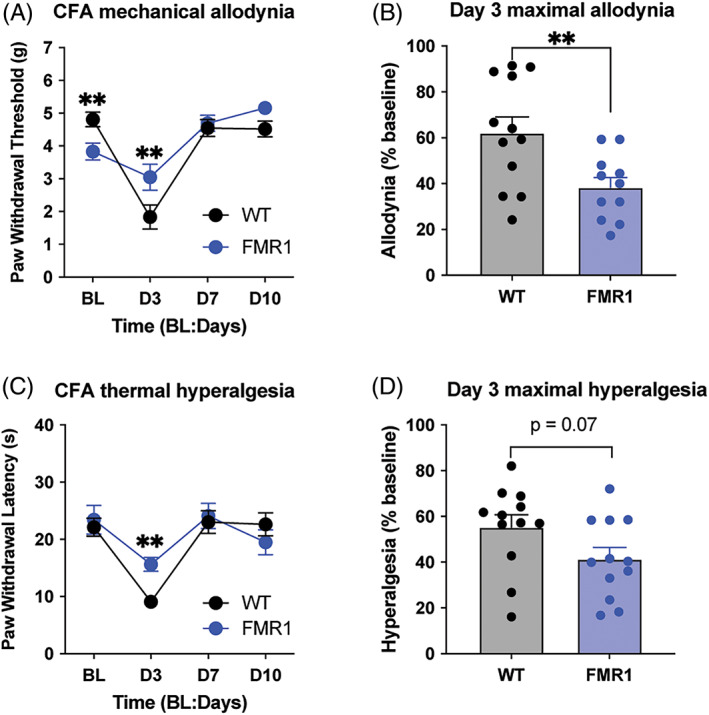
*Fmr1*‐KO mice show altered nociceptive response in chronic inflammatory pain. (A) Basal (BL) mechanical withdrawal thresholds (g) were measured using the von Frey test followed by an intraplantar hind paw injection of complete Freund's adjuvant (CFA). *Fmr1*‐KO (*n* = 12) display lower mechanical thresholds during BL and higher thresholds on Day 3 (D3) post‐CFA injection compared with C57BL/6J (*n* = 12) mice. Mechanical thresholds were not different between the strains on Day 7 (D7) or Day 10 (D10). (B) *Fmr1*‐KO mice display less allodynia when von Frey thresholds on D3 are normalized to BL thresholds for each mouse. (C) BL thermal withdrawal latencies (s) were measured using the radiant heat paw withdrawal followed by an intraplantar hind paw injection of complete Freund's adjuvant (CFA). Thermal thresholds between *Fmr1*‐KO (*n* = 12) and WT (*n* = 12) mice do not differ at BL. Following intraplantar CFA injections, *Fmr1*‐KO mice display longer thermal latencies (s) on D3 but not D7 or D10. (D) *Fmr1*‐KO mice display less hyperalgesia that was trending toward statistical significance (*p* = 0.07). Symbols or bars in all graphs represent mean ± SEM difference; **p < 0.01

Further, mechanical allodynia was significantly reduced following CFA injection (two‐way ANOVA, main effect of strain: *F*_1,22_ = 1.05, *p* = 0.32; main effect of time [RM]: *F*_3,66_ = 35.49, *p* < 0.001; strain × time interaction: *F*_3,66_ = 6.37, *p* < 0.001; Figure 4A) with lower overall maximal allodynia 3 days post‐CFA injection (*t*_22_ = 2.88, *p* = 0.009, Figure 4B). Similarly, thermal hyperalgesia was reduced in *Fmr1*‐KO mice, although not as pronounced as the mechanical phenotype (two‐way ANOVA, main effect of strain: *F*_1,22_==1.68, *p* = 0.28; main effect of time [RM]: *F*_3,66_ = 20.69, *p* < 0.001; strain x time interaction: *F*_3,66_ = 2.81, *p* = 0.046, Figure 4C; maximal hyperalgesia, *t*_22_ = 1.892, *p* = 0.07, Figure 4D).

### 3.3 Reduced sociability and emotional contagion of pain in BTBR and *Fmr1*‐KO mice

**FIGURE 5:**
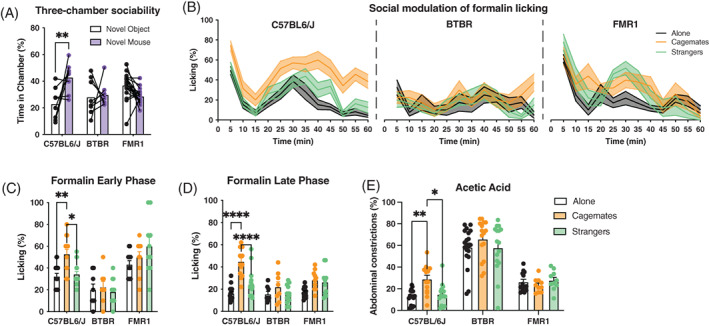
BTBR and *Fmr1*‐KO mice do not exhibit emotional contagion for pain. (A) In the three‐chambered sociability test, C57BL/6J (*n* = 8) show a preference for a novel mouse over a novel object, while BTBR (*n* = 8) and *Fmr1*‐KO (*n* = 18) mice do not show a preference. (B) Graphs show the time course of nocifensive behavior after a 2.5% formalin injection into the plantar hind paw for C57BL/6J, BTBR, and *Fmr1*‐KO mice tested alone or in the presence of a cagemate or stranger. (C,D) Enhanced nocifensive behavior in the early (C) and late phase (D) of the formalin test in C57BL/6J cagemate dyads (*n* = 14) compared with mice tested alone (*n* = 17) or stranger dyads (*n* = 16). Nocifensive behavior is not altered in BTBR, or *Fmr1*‐KO mice tested alone (BTBR, *n* = 8; *Fmr1*‐KO, *n* = 12) or with a cagemate (BTBR, *n* = 8; *Fmr1*‐KO, *n* = 10) or stranger (BTBR, *n* = 12; *Fmr1*‐KO, *n* = 10) during the early or late phase of the formalin test. (E) Enhanced pain behavior in C57BL/6J mice injected with acetic acid (0.9%, intraperitoneal) and paired with cagemate (*n* = 14) compared with mice tested with a stranger (*n* = 16) or alone (*n* = 16). No difference in pain behavior for BTBR, or *Fmr1*‐KO mice tested alone (BTBR, *n* = 20; *Fmr1*‐KO, *n* = 13) or with a cagemate (BTBR, *n* = 16; *Fmr1*‐KO, *n* = 10) or stranger (BTBR, *n* = 16; *Fmr1*‐KO, *n* = 10) during the acetic acid test. Bars or lines in all graphs represent mean ± SEM; *p < 005; **p < 0.01; ***p < 0.001

We previously showed that pain behavior is increased in humans and rodents when conspecifics observe and experience pain with a familiar individual, a phenomenon known as emotional contagion.^32^, ^33^ As ASD mouse models have reduced sociability,^11^ we next wanted to determine whether pain behavior was modulated in the presence of a social partner. To confirm that BTBR and *Fmr1*‐KO mice showed a lack of social preference, we first used the three‐chambered test of sociability. We found that, unlike C57BL6/J mice, BTBR and *Fmr1* KO mice did not spend significantly more time within the chamber containing a novel mouse (two‐way ANOVA, main effect of strain: *F*_2,31_ = 4.09, *p* = 0.03; main effect of chamber: *F*_1,31_ = 1.25, p = 0.27; strain × chamber interaction: *F*_2,31_ = 13.54, *p* < 0.001; Figure 5A). Next, we tested whether the emotional contagion of pain was altered in BTBR and *Fmr1* KO mice. When tested in the presence of a familiar cagemate, C57BL6/J, but not BTBR or *Fmr1*‐KO mice, showed enhanced nociceptive sensitivity during the formalin test (Figure 5B,C,D). The time‐course of nocifensive behavior during the formalin assay is shown in Figure 5B. Analysis of the early phase of formalin (0–10 min) revealed enhanced licking behavior in C57BL6/J mice when tested in the presence of a cagemate and compared with mice tested with a stranger or alone. This effect was not present in BTBR or *Fmr1*‐KO mice (two‐way ANOVA of early phase, main effect of strain: *F*_2,98_ = 32.86, *p* < 0.001; main effect of social condition: *F*_2,98_ = 3.69, *p* < 0.01; strain × social condition interaction; *F*_4,98_ = 3.44, *p* = 0.01; Figure 5C). A similar effect was observed for the late phase of formalin (10–60 min) with C57BL6/J, but not BTBR or *Fmr1*‐KO mice displaying more licking behavior when tested with a cagemate (two‐way ANOVA of late phase, main effect of strain: *F*_2,98_ = 10.58, *p* < 0.001; main effect of social condition: *F*_2,98_ = 19.74, *p* < 0.001; strain × social condition interaction; *F*_4,98_ = 5.16, *p* < 0.001; Figure 5D). As our previous work on the emotional contagion of pain has used the acetic acid writhing test, we also assessed the emotional contagion of pain using acetic acid as the pain stimulus. C57BL6/J mice displayed enhanced pain responses when injected with acetic acid and tested in the presence of a cagemate but not with a stranger partner. This effect was largely absent in BTBR and *Fmr1*‐KO mice, both of which did not show a significant social modulation of pain (two‐way ANOVA, main effect of strain: *F*_2,122_ = 116.8, *p* < 0.001; main effect of social condition: *F*_2,92_ = 2.74, *p* = 0.06; strain × social condition interaction: *F*_4,122_ = 1.8, *p* = 0.13; Figure 5E).

## 4 DISCUSSION

Given that individuals with ASD show altered pain responses, this paper sought to investigate the nociceptive responses of BTBR T^+^ Itpr3^tf^/J (BTBR) and *Fmr1*‐KO mice, two commonly used mouse models of ASD. In the current study, we found that both BTBR T + tf/J and *Fmr1*‐KO models of ASD show mixed pain responses dependent on the sensory modality. BTBR mice showed less sensitivity to mechanical stimulation, while *Fmr1*‐KO mice showed increased mechanical sensitivity. BTBR mice were less sensitive to thermal heat, while *Fmr1*‐KO mice did not display a thermal phenotype. Both BTBR and *Fmr1*‐KO mice showed decreased nocifensive behavior following intraplantar capsaicin and formalin injections, while only BTBR mice were more sensitive on the acetic acid test of visceral sensitivity. In addition, CFA‐induced mechanical allodynia was reduced in BTBR and *Fmr1*‐KO mice, while CFA increased thermal hyperalgesia in BTBR mice. Finally, we also showed that BTBR and *Fmr1*‐KO mice do not display enhanced pain sensitivity when tested with a familiar social partner, an effect we have attributed to the emotional contagion of pain.^32^, ^33^

A previous paper comparing the nociceptive responses of BTBR and *Fmr1‐*KO mice with their respective controls, reported that both strains displayed decreased responsiveness to thermal and electrical stimulation. This same study also reported increased responsiveness to cold stimulation and hypersensitivity to visceral pain stimulation following intraperitoneal injections of acetic acid.^29^ However, the nature of heat responsiveness—increased or decreased—in the BTBR strain may be dependent on the type of thermal test and temperature used. In our study, the thermal phenotype of BTBR mice was only present in the radiant heat paw withdrawal test, while previous reports found a thermal phenotype using the hotplate.^29^, ^40^ Our study set the hotplate temperature to 50°C, while previous papers used a temperature of 55°C.^29^, ^40^ Thus, a lower hotplate temperature may not be sufficient to reveal the thermal heat phenotype, which may only become apparent with greater temperatures. Further, previous studies indicate that *Fmr1*‐KO mice have a mixed thermal phenotype, with some data supporting a decreased thermal phenotype^29^ and some indicating no change.^41^ These findings are also reflected in the human literature, with some ASD individuals reporting enhanced pain sensitivity, decreased sensitivity, or indifference compared with neurotypical controls.^42^

Mechanical behavioral phenotypes have previously been identified for monogenic ASD mouse models, including *Fmr1*,^28^ *Ube3a*,^43^ *Mecp2*,^24^, ^44^ and *Gabrb3*.^44^ Our *Fmr1*‐KO data support these findings and align with increased tactile defensiveness in *Fmr1*‐KO mice, which may interfere with social behavior and interpersonal touch.^28^ Further, *Mecp2* and *Gabrb3* deletion in low‐threshold mechanoreceptors during development, but not adulthood, causes social interaction deficits and anxiety‐like behavior.^44^ This paper also showed that restoring *Mecp2* expression in somatosensory neurons of *Mecp2*‐null mice rescued tactile sensitivity, anxiety‐like behavior, and social interaction deficits, but not memory or motor dysfunction. This suggests that normal touch during critical periods of development is strongly intertwined with brain development and behaviors typically associated with ASD individuals.

Given that touch serves as the primary method of communication in the first years of life, early signs of tactile defensiveness represent a very early indicator of ASD. Indeed, aversion to social touch is among several atypical behaviors observed in infants later diagnosed with autism.^45^ Children with ASD become tense when touched, find touch aversive, and prefer to be touched on their own terms.^4^ Autistic children also report significantly lower pleasantness ratings in response to tactile stimuli than typically developing children.^46^ Hypersensitivity to touch has also been documented in adults with ASD. For example, adults with Asperger's display a significantly lower detection threshold for vibrotactile stimuli and described mild sensations applied to their hand as more “tickly” and intense than control subjects.^47^ In autobiographical accounts, patients with high‐functioning autism have described touch as “an intense feeling” that can be “overwhelming and confusing” and serve as the impetus for social withdrawal.^48^ Conversely, BTBR mice were hyposensitive to mechanical and thermal stimuli, which may be related to the reduced conduction velocity of afferent nerve fibers.^42^

Recent evidence has indicated that chronic pain perception may be implicated in the pathogenesis of poor health outcomes in children with ASD.^49^ The prevalence of pain is twofold higher in ASD children compared with typically developed controls.^50^ However, we find that CFA‐induced mechanical allodynia was reduced for BTBR and *Fmr1*‐KO mice, while thermal hyperalgesia was increased in BTBR mice. Loss of *Fmr1* decreased nociceptive sensitization on the formalin test and delayed the development of neuropathic pain, an effect regulated by the mechanistic target of rapamycin.^27^ Further, inflammatory cytokine production is decreased in human premutation carriers of CGG repeat expansion alleles of between 55 and 200 repeats in the *FMR1* gene and *Fmr1*‐KO mice.^51^ These results coincide with our current results as intraplantar CFA injection (or formalin) may have likely caused a less severe inflammatory response in *Fmr1*‐KO mice. It is a bit unexpected that BTBR mice display increased thermal hyperalgesia, but this may be related to their overall higher thermal latency during baseline testing and a potential floor effect in the magnitude of CFA‐induced hypersensitivity. However, increased thermal pain sensitivity in BTBR mice following CFA injection may be related to desensitization of TRPV1 channels in BTBR tissue.^52^ BTBR mice have alterations in several genes known to regulate synaptic plasticity,^51^ imbalances in excitatory/inhibitory neurotransmission,^53^ impairments in monoaminergic and cholinergic neurotransmission,^54^, ^55^, ^56^, ^57^ all of which may contribute to social behavioral deficits and pain behaviors observed here.

Our study tested only male mice because idiopathic ASD is four times more prevalent in males than in females,^58^, ^59^, ^60^ and fragile X is twice as common in males.^61^ However, this is a limitation of the current work, and future studies should assess pain phenotypes in female ASD mouse models. Although, we have tested female BTBR and *Fmr1*‐KO mice on some of the pain tests used in the current paper (i.e., von Frey, hotplate, and formalin) and have not noticed any obvious sex differences (unpublished data). In addition, the lack of social pain contagion in strangers is only evident in male mice, thus necessitating the sole use of males for the cagemate/stranger comparison.^62^ Further, some papers have tested pain phenotypes in ASD models using both sexes^44^, ^63^; however, no obvious sex differences have emerged. The initial characterization of nociceptive sensitivity in *Fmr1*‐KO mice tested both male and female mice, but no sex differences were reported.^27^ *Shank3* deletion in peripheral mechanosensory neurons leads to tactile hypersensitivity, and region‐specific brain abnormalities with no obvious sex differences reported.^63^ Sex‐specific characterization of prototypical features of ASD has been more commonly investigated than sensory abnormalities in mouse models. For instance, disruption of *Mecp2* in the amygdala of male but not female rats resulted in a significant decrease of juvenile social play behavior.^64^ A careful battery of behavioral tests conducted on female *Fmr1*‐KO mice showed increased repetitive behaviors on the nose‐poke task and enhanced coordination on the accelerating rotarod compared to female WT mice. In contrast, male *Fmr1*‐KOs lacked these behavioral differences.^65^

Combined with the original characterization of pain processing in *Fmr1*‐KO mice that included behavior, anatomy, and electrophysiological responses,^27^ our results and other reports^28^, ^29^ indicate that these mice lack sensitization. Although, in our study we do not find altered visceral sensitivity in *Fmr1*‐KO mice. An increase in visceral pain behavior has been previously reported in *Fmr1*‐KO,^29^ and children with ASD suffer from gastrointestinal problems such as gastroesophageal reflux disease (GERD) and frequent abdominal pain.^66^ Interestingly, the frequency and severity of visceral pain in ASD children have been linked to social withdrawal, stereotype, and hyperactivity compared with children who have no history of frequent GI symptoms.^67^ In a previous study, BTBR mice showed enhanced abdominal constriction behavior compared to C57BL/6J mice.^29^ At the same time, the application of capsaicin and inflammatory mediators increased excitability in jejunum tissue prepared from BTBR mice.^52^ Thus, enhanced visceral sensitivity as observed in the BTBR mouse may partly be due to the enhanced firing of visceral primary afferents following activation by chemical stimuli. Regardless, critical mechanisms of pain regulation in the BTBR model of ASD remain to be uncovered. There are seemingly contradictory findings where these mice were hyposensitive on some tests while hypersensitive on others. These findings recapitulate reports in ASD individuals who show mixed pain responses depending on the sensation.

Finally, the ability of pain to modulate social behavior has been observed in many gregarious species, ranging from rodents to humans, suggesting that a relationship exists between sociability, empathy, and pain perception.^68^, ^69^, ^70^ As pain‐related expressions may communicate one's pain so that care and help may be provided,^71^ we predicted that the asocial BTBR and *Fmr1*‐KO mice would not show increased pain behavior when experiencing pain within a social context. Supporting our prediction, C57BL/6J mice displayed enhanced pain responses on the formalin test when paired with a cagemate, while BTBR and *Fmr1*‐KO mice did not show this type of pain modulation. Similar observations were apparent on the acetic acid test of visceral sensitivity, but the enhanced acetic acid sensitivity of BTBR mice may have created a ceiling effect preventing any further pain enhancement in this strain. However, we are confident in our findings that BTBR mice lack pain contagion because nocifensive responses on the formalin assay were also not modulated by social condition. Previous reports have shown that BTBR mice possess a normal emotional contagion of fear responses,^72^, ^73^ and the transfer of emotional information in BTBR mice may be intact. However, the social transfer of pain and analgesia has recently been shown to depend on the anterior cingulate cortex (ACC)‐to‐nucleus accumbens circuitry, whereas the social transfer of fear, requires ACC projections to the basolateral amygdala.^74^ This would suggest that distinct mechanisms govern the social contagion of emotional information, and a normal fear contagion response in BTBR mice may not necessarily equate with an intact pain contagion response. Human studies have shown that individuals with autism show basic empathetic behaviors such as emotional contagion for pain.^75^ In children with ASD, contagious yawning and laughter are impaired but moderated by familiarity with higher levels of contagion observed between ASD children and their parents.^76^ Further, individuals with ASD score lower on self‐reported measures of empathy. However, they show similar levels of brain activation during the perception of facial pain expressions compared with controls suggesting that reappraisal may lead to a failure of appropriate empathic responding.^75^ In contrast, other studies indicate that the response of an ASD individual to others' pain is dependent on stimulus modality.^10^ The pain behaviors—foot licking or abdominal stretching—used to assess the emotional contagion of pain in BTBR and *Fmr1*‐KO mice are distinct and obvious. Thus, BTBR and *Fmr1*‐KO mice may lack an emotional contagion response due to an inability to perceive the pain expressions from their social partner rather than a true disruption of emotional state sharing.^77^

## CONFLICT OF INTEREST

The authors declare no conflict of interest.

## Data Availability

The data that support the findings of this study are available from the corresponding author upon reasonable request.

## References

[1] American Psychiatric Association . Diagnostic and Statistical Manual of Mental Disorders. 5th ed.; Arlington, VA: American Psychiatric Association; 2013.

[2] Baker AE , Lane A , Angley MT , Young RL . The relationship between sensory processing patterns and behavioural responsiveness in autistic disorder: a pilot study. J Autism Dev Disord. 2008;38(5):867‐875. 17899349 10.1007/s10803-007-0459-0

[3] Brandwein AB , Foxe JJ , Butler JS , Frey HP , Bates JC , Shulman LH . Neurophysiological indices of atypical auditory processing and multisensory integration are associated with symptom severity in autism. J Autism Dev Disord. 2015;45(1):230‐244. 25245785 10.1007/s10803-014-2212-9 PMC4289100

[4] Kern JK , Trivedi MH , Grannemann BD , et al. Sensory correlations in autism. Autism. 2007;11(2):123‐134. 17353213 10.1177/1362361307075702

[5] Rogers SJ , Ozonoff S . Annotation: what do we know about sensory dysfunction in autism? A critical review of the empirical evidence. J Child Psychol Psychiatry. 2005;46(12):1255‐1268. 16313426 10.1111/j.1469-7610.2005.01431.x

[6] Baranek GT , David FJ , Poe MD , Stone WL , Watson LR . Sensory experiences questionnaire: discriminating sensory features in young children with autism, developmental delays, and typical development. J Child Psychol Psychiatry. 2006;47(6):591‐601. 16712636 10.1111/j.1469-7610.2005.01546.x

[7] Peebles KA , Price TJ . Self‐injurious behaviour in intellectual disability syndromes: evidence for aberrant pain signalling as a contributing factor. J Intellect Disabil Res. 2012;56(5):441‐452. 21917053 10.1111/j.1365-2788.2011.01484.x PMC3272540

[8] Duerden EG , Taylor MJ , Lee M , McGrath PA , Davis KD , Roberts SW . Decreased sensitivity to thermal stimuli in adolescents with autism spectrum disorder: relation to symptomatology and cognitive ability. J Pain. 2015;16(5):463‐471. 25704841 10.1016/j.jpain.2015.02.001

[9] Riquelme I , Hatem SM , Montoya P . Abnormal pressure pain, touch sensitivity, proprioception, and manual dexterity in children with autism spectrum disorders. Neural Plast. 2016;2016:1723401. 26881091 10.1155/2016/1723401 PMC4736331

[10] Meng J , Li Z , Shen L . Responses to others' pain in adults with autistic traits: the influence of gender and stimuli modality. PLoS One. 2017;12(3):e0174109. 28319204 10.1371/journal.pone.0174109 PMC5358845

[11] Patterson PH . Modeling autistic features in animals. Pediatr Res. 2011;69(8):34‐40. 21289542 10.1203/PDR.0b013e318212b80f PMC3088489

[12] Balasco L , Provenzano G , Bozzi Y . Sensory abnormalities in autism spectrum disorders: a focus on the tactile domain, from genetic mouse models to the clinic. Front Psych. 2019;10:1016. 10.3389/fpsyt.2019.01016 PMC6997554 32047448

[13] Sato D , Lionel AC , Leblond CS , et al. SHANK1 deletions in males with autism spectrum disorder. Am J Hum Genet. 2012;90(5):879‐887. 22503632 10.1016/j.ajhg.2012.03.017 PMC3376495

[14] Berkel S , Tang W , Trevino M , et al. Inherited and de novo SHANK2 variants associated with autism spectrum disorder impair neuronal morphogenesis and physiology. Hum Mol Genet. 2012;21(2):344‐357. 21994763 10.1093/hmg/ddr470 PMC3276277

[15] Gauthier J , Champagne N , Lafreniere RG , et al. De novo mutations in the gene encoding the synaptic scaffolding protein SHANK3 in patients ascertained for schizophrenia. Proc Natl Acad Sci U S A. 2010;107(17):7863‐7868. 20385823 10.1073/pnas.0906232107 PMC2867875

[16] Eltokhi A , Rappold G , Sprengel R . Distinct phenotypes of Shank2 mouse models reflect neuropsychiatric spectrum disorders of human patients with SHANK2 variants. Front Mol Neurosci. 2018;11:240. 30072871 10.3389/fnmol.2018.00240 PMC6060255

[17] Wang X , Bey AL , Katz BM , et al. Altered mGluR5‐Homer scaffolds and corticostriatal connectivity in a Shank3 complete knockout model of autism. Nat Commun. 2016;7:11459. 27161151 10.1038/ncomms11459 PMC4866051

[18] Ko HG , Oh SB , Zhuo M , Kaang BK . Reduced acute nociception and chronic pain in Shank2^−/−^ mice. Mol Pain. 2016;12:1‐5. 10.1177/1744806916647056 PMC4956181 27145803

[19] Silverman JL , Turner SM , Barkan CL , et al. Sociability and motor functions in Shank1 mutant mice. Brain Res. 2011;1380:120‐137. 20868654 10.1016/j.brainres.2010.09.026 PMC3041833

[20] Han Q , Kim YH , Wang X , et al. SHANK3 deficiency impairs heat hyperalgesia and TRPV1 signaling in primary sensory neurons. Neuron. 2016;92(6):1279‐1293. 27916453 10.1016/j.neuron.2016.11.007 PMC5182147

[21] Jeste SS , Sahin M , Bolton P , Ploubidis GB , Humphrey A . Characterization of autism in young children with tuberous sclerosis complex. J Child Neurol. 2008;23(5):520‐525. 18160549 10.1177/0883073807309788

[22] Sato A , Kasai S , Kobayashi T , et al. Rapamycin reverses impaired social interaction in mouse models of tuberous sclerosis complex. Nat Commun. 2012;3:1292. 23250422 10.1038/ncomms2295 PMC3535343

[23] Downs J , Geranton SM , Bebbington A , et al. Linking MECP2 and pain sensitivity: the example of Rett syndrome. Am J Med Genet A. 2010;152A(5):1197‐1205. 20425824 10.1002/ajmg.a.33314 PMC3913729

[24] Bhattacherjee A , Mu Y , Winter MK , et al. Neuronal cytoskeletal gene dysregulation and mechanical hypersensitivity in a rat model of Rett syndrome. Proc Natl Acad Sci U S A. 2017;114(33):E6952‐E6961. 28760966 10.1073/pnas.1618210114 PMC5565404

[25] Lozano R , Azarang A , Wilaisakditipakorn T , Hagerman RJ . Fragile X syndrome: a review of clinical management. Intractable Rare Dis Res. 2016;5(3):145‐157. 27672537 10.5582/irdr.2016.01048 PMC4995426

[26] Spencer CM , Alekseyenko O , Hamilton SM , et al. Modifying behavioral phenotypes in Fmr1KO mice: genetic background differences reveal autistic‐like responses. Autism Res. 2011;4(1):40‐56. 21268289 10.1002/aur.168 PMC3059810

[27] Price TJ , Rashid MH , Millecamps M , Sanoja R , Entrena JM , Cervero F . Decreased nociceptive sensitization in mice lacking the fragile X mental retardation protein: role of mGluR1/5 and mTOR. J Neurosci. 2007;27(51):13958‐13967. 18094233 10.1523/JNEUROSCI.4383-07.2007 PMC2206543

[28] He CX , Cantu DA , Mantri SS , Zeiger WA , Goel A , Portera‐Cailliau C . Tactile defensiveness and impaired adaptation of neuronal activity in the Fmr1 Knock‐out mouse model of autism. J Neurosci. 2017;37(27):6475‐6487. 28607173 10.1523/JNEUROSCI.0651-17.2017 PMC5511879

[29] Wang L , Almeida LE , Nettleton M , et al. Altered nocifensive behavior in animal models of autism spectrum disorder: the role of the nicotinic cholinergic system. Neuropharmacology. 2016;111:323‐334. 27638450 10.1016/j.neuropharm.2016.09.013 PMC5075237

[30] Daimon CM , Jasien JM , Wood WH 3rd , et al. Hippocampal transcriptomic and proteomic alterations in the BTBR mouse model of autism spectrum disorder. Front Physiol. 2015;6:324. 26635614 10.3389/fphys.2015.00324 PMC4656818

[31] Burket JA , Benson AD , Tang AH , Deutsch SI . Rapamycin improves sociability in the BTBR T(+)Itpr3(tf)/J mouse model of autism spectrum disorders. Brain Res Bull. 2014;100:70‐75. 24295733 10.1016/j.brainresbull.2013.11.005 PMC5581959

[32] Martin LJ , Hathaway G , Isbester K , et al. Reducing social stress elicits emotional contagion of pain in mouse and human strangers. Curr Biol. 2015;25(3):326‐332. 25601547 10.1016/j.cub.2014.11.028

[33] Lidhar NK , Darvish‐Ghane S , Sivaselvachandran S , et al. Prelimbic cortex glucocorticoid receptors regulate the stress‐mediated inhibition of pain contagion in male mice. Neuropsychopharmacology. 2021;46(6):1183‐1193. 33223518 10.1038/s41386-020-00912-4 PMC8115346

[34] Khoutorsky A , Sorge RE , Prager‐Khoutorsky M , et al. eIF2alpha phosphorylation controls thermal nociception. Proc Natl Acad Sci U S A. 2016;113(42):11949‐11954. 27698114 10.1073/pnas.1614047113 PMC5081582

[35] Brenner DS , Golden JP , Gereau RW . A novel behavioral assay for measuring cold sensation in mice. PLoS One. 2012;7(6):e39765. 22745825 10.1371/journal.pone.0039765 PMC3382130

[36] Hargreaves K , Dubner R , Brown F , Flores C , Joris J . A new and sensitive method for measuring thermal nociception in cutaneous hyperalgesia. Pain. 1988;32:77‐88. 3340425 10.1016/0304-3959(88)90026-7

[37] Tjolsen A , Berge OG , Hunskaar S , Rosland JH , Hole K . The formalin test: an evaluation of the method. Pain. 1992;51(1):5‐17. 1454405 10.1016/0304-3959(92)90003-T

[38] Yang M , Silverman JL , Crawley JN . Automated three‐chambered social approach task for mice. Curr Protoc Neurosci. 2011;56(1). 10.1002/0471142301.ns0826s56 PMC4904775 21732314

[39] Tuttle AH , Tansley S , Dossett K , et al. Social propinquity in rodents as measured by tube cooccupancy differs between inbred and outbred genotypes. Proc Natl Acad Sci U S A. 2017;114(21):5515‐5520. 28484016 10.1073/pnas.1703477114 PMC5448193

[40] Silverman JL , Yang M , Turner SM , et al. Low stress reactivity and neuroendocrine factors in the BTBR T+tf/J mouse model of autism. Neuroscience. 2010;171(4):1197‐1208. 20888890 10.1016/j.neuroscience.2010.09.059 PMC2991427

[41] Zhao MG , Toyoda H , Ko SW , Ding HK , Wu LJ , Zhuo M . Deficits in trace fear memory and long‐term potentiation in a mouse model for fragile X syndrome. J Neurosci. 2005;25(32):7385‐7392. 16093389 10.1523/JNEUROSCI.1520-05.2005 PMC6725289

[42] Allely CS . Pain sensitivity and observer perception of pain in individuals with autistic spectrum disorder. ScientificWorldJournal. 2013;2013:916178. 23843740 10.1155/2013/916178 PMC3697411

[43] McCoy ES , Taylor‐Blake B , Aita M , Simon JM , Philpot BD , Zylka MJ . Enhanced nociception in Angelman syndrome model mice. J Neurosci. 2017;37(42):10230‐10239. 28931574 10.1523/JNEUROSCI.1018-17.2017 PMC5647775

[44] Orefice LL , Zimmerman AL , Chirila AM , Sleboda SJ , Head JP , Ginty DD . Peripheral mechanosensory neuron dysfunction underlies tactile and behavioral deficits in mouse models of ASDs. Cell. 2016;166(2):299‐313. 27293187 10.1016/j.cell.2016.05.033 PMC5567792

[45] Baranek GT , Foster LG , Berkson G . Tactile defensiveness and stereotyped behaviors. Am J Occup Ther. 1997;51(2):91‐95. 9124275 10.5014/ajot.51.2.91

[46] Cascio CJ , Lorenzi J , Baranek GT . Self‐reported pleasantness ratings and examiner‐coded defensiveness in response to touch in children with ASD: effects of stimulus material and bodily location. J Autism Dev Disord. 2016;46(5):1528‐1537. 24091471 10.1007/s10803-013-1961-1 PMC3976859

[47] Blakemore SJ , Tavassoli T , Calo S , et al. Tactile sensitivity in Asperger syndrome. Brain Cogn. 2006;61(1):5‐13. 16500009 10.1016/j.bandc.2005.12.013

[48] Cesaroni L , Garber M . Exploring the experience of autism through firsthand accounts. J Autism Dev Disord. 1991;21(3):303‐313. 1938776 10.1007/BF02207327

[49] Tudor ME , Walsh CE , Mulder EC , Lerner MD . Pain as a predictor of sleep problems in youth with autism spectrum disorders. Autism. 2015;19(3):292‐300. 24497628 10.1177/1362361313518994

[50] Whitney DG , Shapiro DN . National prevalence of pain among children and adolescents with autism Spectrum disorders. JAMA Pediatr. 2019;173(12):1203‐1205. 31657837 10.1001/jamapediatrics.2019.3826 PMC6820065

[51] Careaga M , Rose D , Tassone F , Berman RF , Hagerman R , Ashwood P . Immune dysregulation as a cause of autoinflammation in fragile X premutation carriers: link between FMRI CGG repeat number and decreased cytokine responses. PLoS One. 2014;9(4):e94475. 24718368 10.1371/journal.pone.0094475 PMC3981824

[52] Ibrahim H. Investigation of peripheral nerve sensitivity in two animal models of autism spectrum disorder (ASD), Doctoral dissertation, University of Central Lancashire; 2019.

[53] Wei H , Ma Y , Ding C , et al. Reduced glutamate release in adult BTBR mouse model of autism spectrum disorder. Neurochem Res. 2016;41(11):3129‐3137. 27538958 10.1007/s11064-016-2035-5

[54] Gould GG , Hensler JG , Burke TF , Benno RH , Onaivi ES , Daws LC . Density and function of central serotonin (5‐HT) transporters, 5‐HT1A and 5‐HT2A receptors, and effects of their targeting on BTBR T+tf/J mouse social behavior. J Neurochem. 2011;116(2):291‐303. 21070242 10.1111/j.1471-4159.2010.07104.x PMC3012263

[55] Guo YP , Commons KG . Serotonin neuron abnormalities in the BTBR mouse model of autism. Autism Res. 2017;10(1):66‐77. 27478061 10.1002/aur.1665 PMC5518607

[56] Squillace M , Dodero L , Federici M , et al. Dysfunctional dopaminergic neurotransmission in asocial BTBR mice. Transl Psychiatry. 2014;4:e427. 25136890 10.1038/tp.2014.69 PMC4150243

[57] McTighe SM , Neal SJ , Lin Q , Hughes ZA , Smith DG . The BTBR mouse model of autism spectrum disorders has learning and attentional impairments and alterations in acetylcholine and kynurenic acid in prefrontal cortex. PLoS One. 2013;8(4):e62189. 23638000 10.1371/journal.pone.0062189 PMC3634761

[58] Kirkovski M , Enticott PG , Fitzgerald PB . A review of the role of female gender in autism spectrum disorders. J Autism Dev Disord. 2013;43(11):2584‐2603. 23525974 10.1007/s10803-013-1811-1

[59] Lai MC , Lombardo MV , Baron‐Cohen S . Autism. Lancet. 2014;383(9920):896‐910. 10.1016/s0140-6736(13)61539-1 24074734

[60] Halladay AK , Bishop S , Constantino JN , et al. Sex and gender differences in autism spectrum disorder: summarizing evidence gaps and identifying emerging areas of priority. Mol Autism. 2015;6:36. 26075049 10.1186/s13229-015-0019-y PMC4465158

[61] Hunter J , Rivero‐Arias O , Angelov A , Kim E , Fotheringham I , Leal J . Epidemiology of fragile X syndrome: a systematic review and meta‐analysis. Am J Med Genet A. 2014;164A(7):1648‐1658. 24700618 10.1002/ajmg.a.36511

[62] Langford DJ , Crager SE , Shehzad Z , et al. Social modulation of pain as evidence for empathy in mice. Science. 2006;312(5782):1967‐1970. 16809545 10.1126/science.1128322

[63] Orefice LL , Mosko JR , Morency DT , et al. Targeting peripheral somatosensory neurons to improve tactile‐related phenotypes in ASD models. Cell. 2019;178(4):867‐886. 31398341 10.1016/j.cell.2019.07.024 PMC6704376

[64] Kurian JR , Bychowski ME , Forbes‐Lorman RM , Auger CJ , Auger AP . Mecp2 organizes juvenile social behavior in a sex‐specific manner. J Neurosci. 2008;28(28):7137‐7142. 18614683 10.1523/JNEUROSCI.1345-08.2008 PMC2569867

[65] Nolan SO , Reynolds CD , Smith GD , et al. Deletion of Fmr1 results in sex‐specific changes in behavior. Brain Behav. 2017;7(10):e00800. 29075560 10.1002/brb3.800 PMC5651384

[66] McElhanon BO , McCracken C , Karpen S , Sharp WG . Gastrointestinal symptoms in autism spectrum disorder: a meta‐analysis. Pediatrics. 2014;133(5):872‐883. 24777214 10.1542/peds.2013-3995

[67] Chaidez V , Hansen RL , Hertz‐Picciotto I . Gastrointestinal problems in children with autism, developmental delays or typical development. J Autism Dev Disord. 2014;44(5):1117‐1127. 24193577 10.1007/s10803-013-1973-x PMC3981895

[68] Acland EL , Lidhar NK , Martin LJ . Bridging the gap between people and animals: the roots of social behavior and its relationship to pain. In: Vervoort T , Karos K , Trost Z , Prkachin KM , eds. Social and Interpersonal Dynamics in Pain: We Don't Suffer Alone. Cham, Switzerland: Springer International Publishing; 2018:197‐217.

[69] Martin LJ , Tuttle AH , Mogil JS . The interaction between pain and social behavior in humans and rodents. Curr Top Behav Neurosci. 2014;20:233‐250. 24557935 10.1007/7854_2014_287

[70] Sivaselvachandran S , Acland EL , Abdallah S , Martin LJ . Behavioral and mechanistic insight into rodent empathy. Neurosci Biobehav Rev. 2018;91:130‐137. 10.1016/j.neubiorev.2016.06.007 27311631

[71] Craig KD . Social communication model of pain. Pain. 2015;156(7):1198‐1199. 26086113 10.1097/j.pain.0000000000000185

[72] Keum S , Park J , Kim A , et al. Variability in empathic fear response among 11 inbred strains of mice. Genes Brain Behav. 2016;15(2):231‐242. 26690560 10.1111/gbb.12278

[73] Keum S , Kim A , Shin JJ , Kim JH , Park J , Shin HS . A missense variant at the Nrxn3 locus enhances empathy fear in the mouse. Neuron. 2018;98(3):588‐601. 29681532 10.1016/j.neuron.2018.03.041

[74] Smith ML , Asada N , Malenka RC . Anterior cingulate inputs to nucleus accumbens control the social transfer of pain and analgesia. Science. 2021;371(6525):153‐159. 33414216 10.1126/science.abe3040 PMC7952019

[75] Hadjikhani N , Zurcher NR , Rogier O , et al. Emotional contagion for pain is intact in autism spectrum disorders. Transl Psychiatry. 2014;4:e343. 24424389 10.1038/tp.2013.113 PMC3905223

[76] Helt MS , Fein DA , Vargas JE . Emotional contagion in children with autism spectrum disorder varies with stimulus familiarity and task instructions. Dev Psychopathol. 2020;32(1):383‐393. 30924430 10.1017/S0954579419000154

[77] Prochazkova E , Kret ME . Connecting minds and sharing emotions through mimicry: a neurocognitive model of emotional contagion. Neurosci Biobehav Rev. 2017;80:99‐114. 28506927 10.1016/j.neubiorev.2017.05.013

